# Design and Field Assessment of a Pressurized Driving-Down Air Multilevel Sampler for Depth-Discrete Groundwater Monitoring in NAPL Impacted Wells

**DOI:** 10.3390/s26123788

**Published:** 2026-06-14

**Authors:** Giuseppe Passarella, Rita Masciale, Antonio Di Fazio, Costantino Masciopinto

**Affiliations:** 1 Water Research Institute of the National Research Council of Italy, 70132 Bari, Italy; costantino.masciopinto@cnr.it; 2 Water Resources Commission, Regional Order of Geologists of Puglia, 70125 Bari, Italy; antoniodifazio@tiscali.it

**Keywords:** groundwater monitoring sensors, subsurface environmental sampling, VOC-preserving sampling technologies, pressure-driven water extraction, NAPL-impacted aquifers, karst and fractured media instrumentation, multilevel sampling systems, hydraulic flow modeling

## Abstract

**Highlights:**

**What are the main findings?**

- A novel pressure-driven multilevel sampler was developed to collect depth-discrete groundwater samples below floating NAPL layers.
- Field testing confirmed stable hydraulic performance, low disturbance of the water column, and compatibility with VOC-sensitive analyses.

**What are the implications of the main findings?**

- The system provides a practical solution for groundwater monitoring in fractured and contaminated aquifers where conventional methods may be unreliable.
- The proposed technology can improve high-resolution contaminant characterization and support long-term environmental monitoring strategies.

**Abstract:**

This study presents the development and field testing of a Pressurized Driving-Down Air Multilevel Sampler (PDA-MLS), an integrated groundwater sampling device designed for depth-discrete sampling in boreholes affected by floating non-aqueous phase liquids (NAPLs). Conventional sampling methods—such as low-flow pumps, bailers, and packer-isolated systems—often fail under these conditions due to limited accessibility, cross-contamination, or disturbance of the water column. The proposed system addresses these limitations through a controlled pressurized-gas actuation mechanism that transfers groundwater from multiple PTFE-membrane chambers installed at discrete depths. This configuration enables low-disturbance sampling below floating contaminant layers. The use of chemically inert materials (stainless steel and PTFE) minimizes sampling artifacts and ensures compatibility with volatile organic compound (VOC) analyses. A simplified hydraulic conceptual framework describing inflow, outflow, and pressure-driven displacement was developed to support purge-duration estimation and operational parameter definition. The device was tested in a 90 m deep fractured limestone aquifer contaminated by tetrachloroethylene (PCE), where floating hydrocarbons limited the applicability of conventional sampling techniques. Field testing showed stable discharge conditions (~145–160 mL/min), repeatable sampling cycles, and successful collection of depth-discrete groundwater samples under the investigated site conditions. No evidence of sampler-related hydrocarbon entrainment was observed in the collected samples within the analytical detection limits of the adopted laboratory methods. To the authors’ knowledge, the PDA-MLS represents one of the few groundwater sampling systems specifically designed to combine low-disturbance multilevel sampling with operation in wells affected by floating NAPL. These features make it a promising tool for environmental monitoring, high-resolution characterization of fractured aquifers, and long-term assessment of contaminated sites.

## 1. Introduction

Groundwater monitoring plays a central role in environmental protection and contamination assessment, particularly in fractured and karst aquifers, where flow heterogeneity complicates sampling strategies [1,2]. Conventional sampling techniques recommended by regulatory agencies such as the U.S. EPA [3] typically rely on submersible pumps or bailers [4], which may induce vertical mixing, disturb the water column, and promote aeration and loss of volatile compounds [5,6,7]. These limitations become critical in boreholes affected by floating non-aqueous phase liquids (NAPLs), where instrument immersion is either impractical or strongly discouraged due to the risk of cross-contamination [8].

Previous studies have highlighted the limitations of mechanical pumping systems in maintaining sample integrity under such conditions. Low-flow and inertial pumping methods may generate localized turbulence and disturb salinity and VOC profiles [9,10]. Multilevel sampling systems, such as those originally proposed by [11,12] and further developed by [13], enable depth-discrete sampling but often require complex installations involving dedicated tubing, packers, or pre-installed liners. Similarly, FLUTe^®^ systems (Flexible Liner Underground Technologies, Santa Fe, NM, USA) systems [14,15] provide high-resolution vertical sampling but require clean borehole conditions and stable hydraulic regimes, which are rarely encountered in contaminated or NAPL-impacted environments.

Recent developments in environmental monitoring technologies have explored pressure-driven sampling approaches based on pneumatic actuation sampling approaches capable of operating under challenging hydraulic conditions. Gas-driven extraction systems have been successfully applied in geothermal and CO_2_ storage wells [16], demonstrating the feasibility of pressure-controlled sampling. However, these systems are typically designed for large-diameter industrial boreholes and are not easily adaptable to standard environmental monitoring wells. These limitations highlight a technological gap in the availability of compact, chemically inert, and mechanically stable multilevel sampling systems capable of operating below floating NAPL layers while minimizing disturbance of groundwater conditions. Commonly used approaches still face challenges in simultaneously achieving high vertical resolution, minimizing hydraulic disturbance, and avoiding sampling bias in heterogeneous environments. These limitations are particularly relevant in fractured aquifers characterized by strong vertical heterogeneity. In parallel, advances in hydrogeological modeling and environmental monitoring have emphasized the importance of high-resolution vertical profiling for understanding contaminant transport, salinity gradients, and aquifer dynamics [17,18,19].

Within this context, the PDA-MLS builds upon existing multilevel concepts but introduces a key advancement through the integration of pressure-driven actuation with controlled chamber-based sampling. This configuration enables depth-discrete sampling without continuous flow extraction, thereby reducing hydraulic disturbance and minimizing the risk of cross-contamination between sampling intervals. Therefore, the novelty of the proposed system lies not in the individual components, but in their combined operation, which allows controlled, low-disturbance, multilevel sampling under conditions where conventional approaches are limited.

Existing groundwater sampling techniques can be compared based on a set of key performance criteria, including vertical resolution, sampling bias, recovery efficiency, and the degree of hydraulic disturbance induced during operation. These parameters are particularly critical in fractured and NAPL-impacted aquifers, where small-scale heterogeneities and phase distribution significantly influence measured concentrations. In this context, low-flow purging methods may introduce vertical mixing and sampling bias, while passive samplers rely on equilibrium conditions that may not adequately represent dynamic concentration profiles. Multilevel systems improve vertical resolution but often involve increased installation complexity and potential operational constraints.

To provide a structured comparison with existing groundwater sampling approaches, a comprehensive evaluation of key performance criteria is presented and discussed later in the manuscript, where the proposed system is discussed in relation to currently available technologies. Parallel efforts in the Apulia Region have underscored the role of multi-parameter chemical datasets in distinguishing natural and anthropogenic groundwater signatures [20,21,22]. These advancements collectively demonstrate the need for a multilevel sampling device that can obtain depth-discrete groundwater samples under conditions affected by surface NAPL obstructions.

To address these challenges, this study presents a Pressurized Driving-Down Air Multilevel Sampler (PDA-MLS), an integrated groundwater sampling system based on pressure-driven actuation. The device is designed to collect depth-specific groundwater samples without disturbing the water column or interacting with floating contaminants. The system combines chemically inert materials with a controlled pressure-driven mechanism to enable controlled and repeatable sampling under complex hydrogeological conditions.

To the authors’ knowledge, this system represents one of the few multilevel groundwater sampling approaches specifically designed to operate effectively in wells affected by floating NAPL while maintaining low-disturbance conditions and while minimizing hydraulic disturbance and sampling artifacts.

The objectives of this study are to: (i) present the design and operating principles of the PDA-MLS; (ii) describe its material selection and simplified hydraulic conceptual framework; (iii) evaluate its performance through field testing in a fractured aquifer affected by hydrocarbon contamination; and (iv) assess its potential applicability for environmental monitoring and groundwater sensing applications.

## 2. Materials and Methods

### 2.1. Overview of Multilevel Sampling Technologies

Environmental monitoring in complex aquifers has stimulated the development of numerous multilevel sampling systems over the last four decades. Early tools, such as the multilevel samplers by [11,12], introduced the concept of discrete-depth sampling through isolated intervals within a single borehole. Subsequent advancements included Continuous Multichannel Tubing (CMT) type systems [13], passive diffusion samplers, and gas-driven sampling devices developed for geothermal and CO_2_-storage wells [16]. Despite their utility, several limitations persist: (i) dependence on pre-installed liners; (ii) limited operating pressure tolerance; (iii) susceptibility to stagnant internal volumes; and (iv) incompatibility with wells affected by floating NAPLs. Table 1 presents a selection of commonly used discrete-depth groundwater sampling tools, detailing their construction materials, extraction mechanisms, suitable aquifer conditions, and maximum effective sampling depths. The cited references correspond to the original studies that describe each tool’s design, performance characteristics, or field applications. These constraints support the development of alternative pressure-driven samplers capable of operating under complex hydrogeological and NAPL-impacted conditions.

### 2.2. PDA-MLS Design and Operating Principle

**Figure 1:**
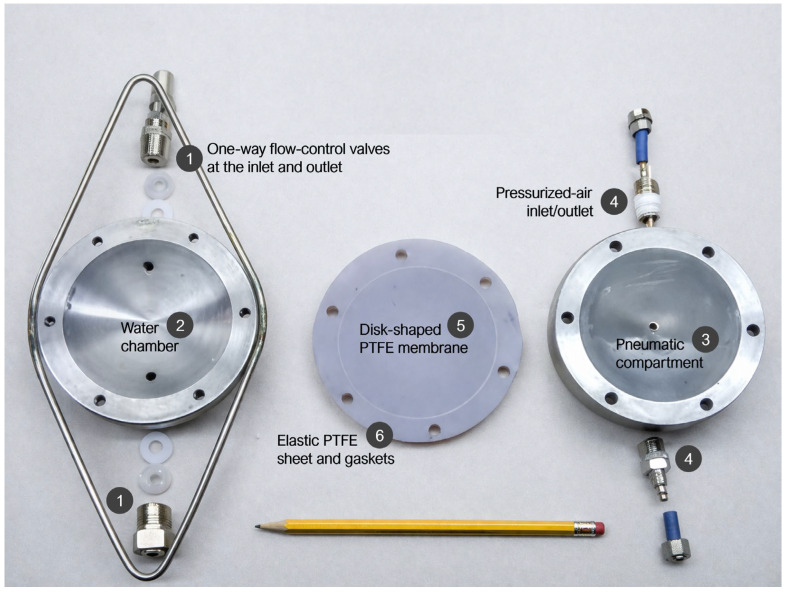
Components of the membrane-based MLS system.

The PDA-MLS was engineered as a mechanically stable, chemically inert, and field-deployable system for sampling groundwater beneath floating hydrocarbons. Each sampler consists of two stainless steel disks enclosing a PTFE membrane that separates a water chamber from an air/pressure chamber (Figure 1). During operation, a controlled pressurized-gas pulse deforms the membrane and expels groundwater upward through individual tubing lines. Once the pulse ceases, natural hydrostatic pressure refills the chamber, establishing a cyclic inflow/outflow process. This configuration eliminates the need for pumps, bailers, or packers, thereby reducing mechanical disturbance and preventing contact with NAPL layers at the water table.

The PDA-MLS was designed to withstand pressures up to 1.0 MPa, enabling deployment in deep wells (up to 90 m in the current configuration). The sampler body, constructed from stainless steel with high chemical resistance, ensures negligible reactivity with dissolved metals or VOCs. PTFE, subjected to extensive testing in groundwater monitoring applications [6], was selected for its minimal adsorptive behavior and strong resistance to degradation.

**Figure 2:**
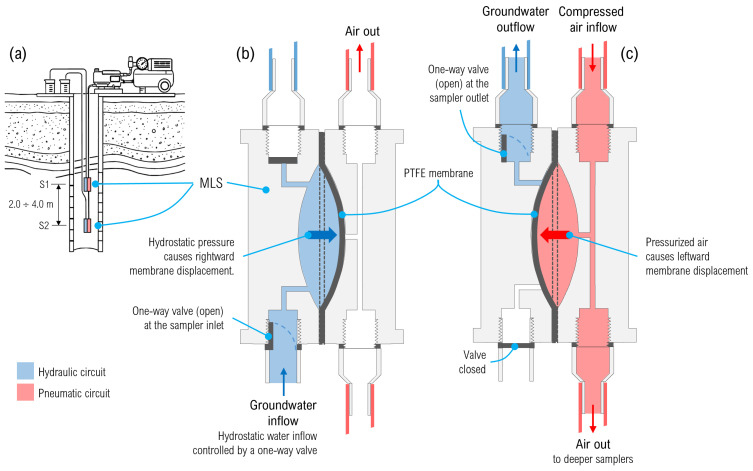
Overview of the MLS installation, and functional schematic representation of the PDA-MLS operating cycle: (**a**) Field installation layout of the PDA-MLS system. (**b**) Filling phase: groundwater enters the hydraulic chamber due to the local hydraulic head gradient while the pneumatic compartment remains depressurized. (**c**) Pressurization and discharge phase: compressed air induces membrane deformation and pressure-driven displacement of groundwater toward the surface collection system (Not to scale.). Blue and red colors identify the hydraulic and pneumatic circuits, respectively, while the arrows indicate the direction of water and compressed-air flow during the operating cycle.

The PDA-MLS operating cycle consists of two main phases, schematically illustrated in Figure 2: (i) chamber filling and (ii) pressure-driven sample discharge. During the filling phase (Figure 2a), groundwater enters the water chamber through the inlet line due to the hydraulic head difference between the aquifer and the initially depressurized chamber, while the pneumatic compartment remains at atmospheric pressure. During the discharge phase (Figure 2b), compressed air is introduced into the pneumatic compartment, inducing membrane deformation and increasing pressure within the hydraulic chamber. The resulting pressure gradient drives groundwater upward through the outlet tubing toward the surface collection system. The flexible membrane physically separates the pneumatic and hydraulic compartments throughout operation, preventing direct contact between compressed air and groundwater. During the extraction phase, a positive pressure gradient is maintained to minimize backflow and uncontrolled mixing within the sampling line. Under simplified assumptions, the discharge process can be qualitatively interpreted as pressure-driven flow governed by the pressure difference between the pneumatic compartment and the hydraulic head within the outlet line.

### 2.3. Study Area: Geological and Hydrogeological Setting

**Figure 3:**
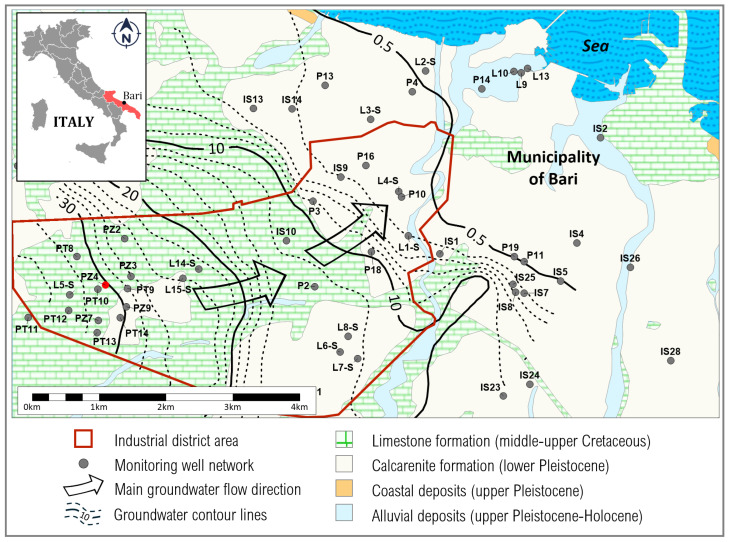
Location map of the Modugno–Bari fractured karst aquifer area showing geological units, monitoring wells, coastal boundary, groundwater potentiometric surface above sea level, and inferred flow directions. The red dot identifies the PZ4 monitoring well selected for PDA-MLS field testing.

The field evaluation of the PDA-MLS was carried out in the industrial district of Bari–Modugno (Apulia, Southern Italy), an area extensively studied for its fractured carbonate aquifer system and complex contamination patterns (Figure 3). The region is underlain by a thick Cretaceous limestone–dolostone sequence (Calcare di Bari Formation), characterized by pronounced karstification, fracture networks, and irregular conduit geometries. Overlying Quaternary deposits, including calcarenites and alluvial sediments, locally modify recharge pathways and introduce permeability contrasts [30,31].

Groundwater flow predominantly occurs within sub-horizontal fracture systems, exhibiting anisotropic hydraulic conductivities typically ranging from 10^−3^ to 10^−4^ m/s. The aquifer is recharged both by regional infiltration (30% of mean annual rainfall) and local contributions through minor karst depressions and ephemeral channels known as “lame” [32]. Numerous studies—such as [18,20,33,34,35]—have documented the variability of groundwater salinity, natural background levels, and the influence of anthropogenic pollutants in this region.

The site selected for PDA-MLS deployment (well PZ4) exhibits a 20–30 cm thick floating hydrocarbon layer attributed to historical industrial spills. Conventional sampling methods proved unsuitable due to the necessity of avoiding contact with the NAPL layer and preventing disturbance of the underlying water column. The fractured nature of the aquifer further complicates sampling by encouraging vertical flows within the borehole annulus, especially during tool insertion.

Hydro-chemical surveys conducted in the study area have consistently identified tetrachloroethylene (PCE) as the primary contaminant, originating from dense non-aqueous phase liquid (DNAPL) ganglia in shallow subsoil layers [36]. Earlier investigations [37] and tracer-based hydraulic characterizations [17] demonstrate the presence of multiple fracture conduits that influence contaminant migration. The concurrence of floating hydrocarbons, fracture-controlled groundwater flow, and volatile contaminants makes the site particularly well-suited for evaluating the PDA-MLS under demanding field conditions.

### 2.4. Field Deployment and Experimental Configuration

The PDA-MLS system was installed in well PZ4 (Figure 3) using two samplers positioned at depths of 57.5 m (S_1_) and 60 m (S_2_) below ground level, separated by a 2.5 m vertical interval. During installation, low-pressure gas pulses (<0.05 MPa) were applied to minimize backflow and potential cross-contamination (Figure 2). Once positioned, a full purge cycle was conducted to remove stagnant water from sampler chambers and tubing, based on the simplified hydraulic framework and adjusted through field observation of discharge conditions.

The surface setup included a pressurized-gas compressor (1.0 MPa capacity), a programmable solenoid valve, and individual tubing lines for each sampler. Pressure pulses were administered at 15 s intervals, expelling approximately 42.6 mL of groundwater per cycle. During field operations, pneumatic pressure was regulated using a controlled compressed-air supply and periodically checked throughout the sampling cycles to maintain stable operating conditions. Tubing connections and sealing components were inspected prior to deployment to minimize leakage and pressure losses. Although no formal calibration procedure was implemented, discharge consistency was qualitatively assessed through repeated filling and extraction cycles performed under comparable operating conditions.

Water samples were collected into airtight glass vials and immediately stored in portable refrigerated containers for transfer to the analytical laboratory of the Regional Environmental Protection Agency (ARPA Puglia), where major cations/anions and VOCs were analyzed using standard EPA methods [38,39].

### 2.5. Simplified Hydraulic Framework and Operational Assessment

A simplified hydraulic conceptual framework based on Bernoulli’s principle [40] was adopted as a supporting tool to estimate the order of magnitude of inflow and outflow rates and to define minimum operational times for filling and purging. Using the geometric characteristics of the inlet section and the hydraulic head conditions, the inflow rate was estimated to be approximately $q_{in}=166\;mL\;{min}^{-1}$. Based on the sampler chamber volume (Sc=42.6 mL), the corresponding filling time was approximately $t_{in}\approx 15\;s$. Similarly, the theoretical outflow rate $q_{out}$ during air injection was estimated to be about 162 mL min^−1^ with a corresponding discharge time $t_{out}\approx 16\;s$.

Following MLS installation, an initial purging operation cycle was performed to remove stagnant water from the samplers and tubing system. The total internal volume was estimated from a tubing capacity of 15.7 mL m^−1^, yielding approximately 1000 mL over 60 m, the depth of the lower sampler. Based on the estimated outflow rate, the minimum time required to extract 1000 mL per sampler was slightly larger than 6 min. To ensure complete flushing, including the internal tubing volume, a conservative duration of 10 min was adopted (Table 2). During this phase, continuous air injection forced water upward through the system, progressively replacing stagnant water with formation groundwater.

After purging, the system was operated through repeated sampling cycles. Each cycle consisted of a filling phase driven by natural hydraulic head and a discharge phase induced by compressed air injection. A compressed-air injection interval of 15 s was adopted to ensure complete filling of the sampler chamber. Sampling continued until the required volume was collected. In this study, four 1.0 L bottles per sampler were obtained in approximately 30 min ($t_{s}\approx 6.9\;min/L$), corresponding to an average discharge $q_{meas}$ of about 145 mL min^−1^. Sampling rates remained reasonably consistent over repeated cycles, confirming system repeatability.

The proposed framework should be interpreted as a simplified operational estimation approach intended to support qualitative interpretation of purge duration and pressure-driven discharge behavior during field deployment, rather than as a fully predictive hydraulic model. The framework assumes simplified pressure conditions, idealized flow behavior within the tubing system, and negligible transient hydraulic losses associated with valve operation, local turbulence, and minor head fluctuations. Consequently, the estimated discharge values should be interpreted as approximate operational estimates dependent on site-specific hydraulic and pneumatic conditions.

The comparison between estimated and observed discharge values is intended as an operational consistency assessment rather than formal hydraulic model validation. The estimated outflow (≈162 mL min^−1^) and the observed discharge (≈145 mL min^−1^) differed by approximately 10%, which is considered reasonably compatible with the simplified assumptions underlying the framework and with expected operational variability under field conditions. Similarly, the estimated minimum extraction time (≈6.1 min per liter) and the observed sampling time (≈6.9 min) showed satisfactory agreement for operational purposes. The simplified framework enabled definition of purge duration and operational cycling parameters without the need for substantial iterative field adjustments. The obtained flow rates and operating conditions are also generally consistent with values reported for pneumatic and low-flow groundwater sampling systems [41,42,43]. Additional qualitative observations from controlled tests conducted in the L3s borehole (Figure 3) showed comparable operational behavior. (Figure 3).

## 3. Results from Field Testing of the PDA-MLS System

Field trials of the PDA-MLS were conducted at well PZ4, located within the fractured carbonate aquifer of the Bari–Modugno industrial district (Figure 3). The primary objective of these tests was to evaluate the sampler’s operational stability, hydraulic performance, sample integrity, and suitability for extracting depth-specific groundwater in the presence of floating NAPL. The results suggest that the PDA-MLS can generate reliable depth-discrete groundwater samples under field conditions where conventional approaches may present operational limitations.

During installation, both samplers (S_1_ at 57.5 m and S_2_ at 60 m) (Figure 2) reached their designated depths without mechanical obstruction or buoyancy-related misalignment, indicating optimal weight distribution and structural rigidity of the stainless-steel bodies. Hydrostatic refilling occurred consistently after each pressurized-gas pulse, with refill times reasonably compatible with the simplified hydraulic estimates. Measured upward discharge averaged ~160 mL/min and remained reasonably stable over repeated operational cycles, indicating consistent pressure-driven actuation under the investigated field conditions. This level of reproducibility demonstrates reliable control of pressure-driven actuation under tested conditions and suggests efficient pressure-driven flow within the sampling system.

One of the most relevant operational outcomes concerns the apparent hydraulic separation between the floating diesel-like hydrocarbon layer and the sampled groundwater. The PDA-MLS allowed sampling beneath the NAPL interface without evident downward perturbation of the immiscible layer during field operations. No visible evidence of hydrocarbon entrainment or anomalous contamination attributable to sampler-induced disturbance was observed in the collected groundwater samples. The consistent absence of hydrocarbons across multiple cycles suggests that the system minimizes vertical mixing and reduces the likelihood of contaminant entrainment from the water-table region.

Chemical analyses of samples collected from S1 and S2 were primarily intended to verify the absence of evident sampler-induced disturbance, volatilization artifacts, or NAPL entrainment during depth-discrete sampling operations, rather than to provide a comprehensive hydrochemical characterization of the investigated aquifer. Selected chemical indicators and NAPL-related parameters are reported in Table 3. Measured electrical conductivity values (724–738 µS/cm) and selected dissolved constituents showed limited but observable variability between sampling depths, suggesting localized hydrochemical heterogeneity consistent with the fractured nature of the aquifer system. TCE concentrations remained low (0.06–0.1 µg/L), in agreement with previous investigations indicating that the PZ4 well is not a major release point for the contaminant plume. The PDA-MLS system enabled collection of depth-discrete groundwater samples under conditions where the presence of floating NAPL limited the applicability of conventional pumping-based approaches. The observed variability between sampling intervals suggests that the system may support investigation of localized vertical heterogeneity within fractured carbonate aquifers, although additional multi-site validation would be required to further assess the broader applicability of the approach.

## 4. Discussion

This section provides a critical evaluation of the PDA-MLS system, focusing on its performance, advantages related to existing technologies, operational limitations, and potential future developments.

The PDA-MLS demonstrated several operational characteristics that may provide advantages compared to conventional groundwater sampling approaches under complex hydrogeological conditions.

First, the system’s pressure-driven actuation achieves controlled, low-disturbance extraction that helps preserve the natural stratification of groundwater. This feature is particularly important in fractured aquifers, where local hydraulic heads and fracture connectivity cause rapid vertical flow fluctuations. Unlike bailers or pumps, the PDA-MLS exerts minimal mechanical stress on the water column, thus reducing the likelihood of disturbance of floating hydrocarbons, fine sediments, and dissolved gases.

Second, the device exhibits high chemical inertness due to its stainless-steel and PTFE components. No evident leaching or sorption effects were observed for the measured chemical species under the investigated operating conditions. This is especially relevant in VOC-sensitive investigations, where contact with reactive materials can bias analytical results. The short residence time of water inside the sampler further minimizes interaction with sampler surfaces.

Third, the PDA-MLS showed stable mechanical operation during field deployment. Its fixed geometry facilitates deployment in challenging well conditions, including irregular borehole walls, partial clogging, and the presence of NAPL layers. The ability to generate consistent discharge rates even at depths approaching 60 m suggests that the system can maintain reliable pressure actuation and overcome hydrostatic loads.

Fourth, the system’s modularity and ease of assembly make it suitable for field teams with varying levels of technical expertise. Unlike packer-based equipment, the PDA-MLS does not require the same level of specialized training typically associated with packer-based hydraulic isolation systems. Once installed, the programmable solenoid valve manages all pressurized-gas pulses without manual intervention, allowing technicians to focus on sample handling and data recording.

To provide a structured comparison with existing methodologies, a comprehensive evaluation based on key performance criteria, including vertical resolution, sampling disturbance, and operational complexity, is presented in Table 4, where the proposed PDA-MLS system is critically compared with currently available technologies. The qualitative comparison reported in Table 4 is based on published literature, operational considerations, and field observations.

Table 4 suggests that the PDA-MLS combines low-disturbance sampling, chemical inertness, and operational reliability under complex hydrogeological conditions. Unlike conventional techniques, which are often affected by mixing, aeration, or limited applicability in the presence of NAPL, the proposed system may support reproducible and depth-resolved groundwater sampling under the investigated operating conditions. This capability is particularly relevant for fractured aquifers, where accurate characterization of vertical contaminant distribution is essential for reliable hydrogeological modeling and risk assessment.

Despite the promising performance of the PDA-MLS system, several limitations should be acknowledged, also indicating directions for future development. The present study is based on field testing conducted under a limited set of hydrogeological and operational conditions within a single contaminated fractured aquifer site. Consequently, the proposed hydraulic framework and discharge estimates should be interpreted as simplified operational approximations rather than fully predictive hydraulic assessments.

Operational uncertainties associated with pneumatic pressure regulation, tubing friction losses, local hydraulic head fluctuations, and manual discharge measurements may have contributed to the observed differences between estimated and measured discharge values. Although repeated sampling cycles showed reasonably stable operating behavior under the investigated field conditions, the limited number of measurements does not allow a rigorous statistical assessment of system variability and long-term reproducibility. Additional testing under different hydrogeological settings and controlled laboratory calibration would therefore be beneficial to further quantify operational uncertainty, repeatability, and long-term system performance. Similarly, chemical measurements may also be affected by uncertainties associated with sample handling, minor volatilization processes, material-related adsorption effects, and local concentration variability within fractured aquifer environments.

Beyond the methodological and operational uncertainties discussed above, an additional practical limitation concerns dependence on external pressure-control systems and compressed gas sources. While the current design relies on a surface compressor, this requirement may limit applicability in low-resource or remote field conditions. In such cases, alternative solutions such as lightweight gas cartridges or battery-powered micro-compressors could be considered to improve portability and enable deployment in off-grid environments, including emergency response or remote monitoring networks. In addition, the need for trained personnel and careful operational setup and pressure regulation procedures may increase operational complexity compared to simpler passive sampling approaches.

A further limitation relates to the maximum achievable operating depth. With the current configuration (approximately 1.0 MPa compressor), the system is limited to depths of about 90 m. The adoption of higher-pressure compressors, optimized tubing diameters, or reinforced materials could extend the operational range to greater depths (e.g., up to 150 m), although this would require careful management of pressure losses and system robustness. Furthermore, the applicability of the system may be constrained in very low-permeability formations, where recharge rates are insufficient to sustain repeated sampling cycles without inducing measurable hydraulic disturbance. Similarly, the relatively complex tubing and multichambered configuration may affect scalability and increase installation time, particularly in deep or narrow boreholes. Finally, although the system is designed to minimize disturbance, pressure-driven actuation may mobilize contaminants under certain site-specific conditions. For this reason, its application should be carefully evaluated depending on local hydrogeological characteristics.

Another important aspect is the current absence of embedded real-time sensors within each sampling unit. The integration of miniature pressure, temperature, or electrical conductivity probes would enable continuous monitoring during sampling, providing additional insight into borehole dynamics and sample stabilization. Such developments would represent a significant step toward next-generation, sensor-integrated groundwater monitoring systems.

The PDA-MLS system is particularly suited for applications requiring depth-discrete groundwater sampling in complex hydrogeological settings, such as fractured aquifers and NAPL-impacted sites, where maintaining stratification and avoiding cross-contamination are essential. An important direction for future development concerns the reduction in sampling time and the potential transition toward semi-continuous or real-time monitoring. In the current configuration, sampling duration is influenced by pneumatic actuation cycles, chamber volumes, and the need to ensure minimal hydraulic disturbance during operation. Future improvements could focus on optimizing pressure regulation, reducing internal dead volumes, and integrating automated control systems and inline sensors. These advances could significantly enhance temporal resolution and allow for quasi-real-time monitoring capabilities. Additionally, system miniaturization and modular design may facilitate broader deployment in long-term monitoring networks and increase adaptability to different well configurations and site conditions.

## 5. Conclusions

This study presented the development, operating principle, and field validation of a PDA-MLS designed for depth-discrete groundwater sampling in monitoring wells affected by floating non-aqueous phase liquids (NAPLs). The system was successfully deployed in a fractured carbonate aquifer, where it demonstrated stable and repeatable hydraulic performance, enabling groundwater sampling beneath a hydrocarbon layer without evident cross-mixing or sample aeration under the investigated field conditions. The use of stainless-steel housings combined with PTFE membranes ensured high chemical inertness and mechanical robustness, supporting reliable analyses of volatile and dissolved constituents.

Compared with conventional groundwater sampling techniques—including low-flow pumps, bailers, and packer-isolated systems—the PDA-MLS exhibited three key advantages: (i) the capability to operate effectively in wells where floating contaminants limit or prevent the use of traditional instruments; (ii) minimal hydraulic disturbance of the water column, helping preserve the natural vertical stratification of groundwater chemistry, particularly in fractured aquifers; and (iii) reduced operational complexity compared with packer-based systems, as the system does not require packers, dedicated well completions, or complex pumping systems. These characteristics provide a practical and efficient solution for multilevel groundwater sampling in complex hydrogeological environments.

The simplified hydraulic framework developed in this study provided operational support for estimating purge volumes and actuation intervals, with measured discharge rates showing reasonable agreement with theoretical estimates under the investigated field conditions.

This may support pre-planning of sampling strategies, minimizing field time while ensuring representativeness. The field results revealed depth-dependent hydrochemical patterns that may be more difficult to resolve using conventional techniques, highlighting the diagnostic value of the PDA-MLS for investigating contaminant distribution in fractured and karst aquifers.

The results further demonstrate that the PDA-MLS is particularly suitable for contaminated industrial environments affected by fuel spills, chlorinated solvents, and mixed-phase pollution. Its ability to minimize hydraulic disturbance under controlled operating conditions enables reliable sampling beneath floating hydrocarbons, even where conventional methods are impractical or prone to bias. In fractured and karst aquifers, the system provides a reliable tool for delineating discrete flow paths and vertical chemical gradients, offering valuable support for conceptual and numerical models of contaminant transport.

From an operational perspective, the PDA-MLS offers immediate practical benefits for environmental monitoring and site management. It enables the recovery of undisturbed samples beneath floating NAPL interfaces, supports the reconstruction of high-resolution vertical concentration profiles, reduces uncertainty in conceptual site models, and improves the reliability of remediation design. Its modularity, ease of deployment, and chemical compatibility reduce training requirements and minimize the risk of sampling artifacts, particularly in investigations involving volatile organic compounds.

Despite its demonstrated effectiveness, the PDA-MLS presents some operational limitations that define directions for further development. The current depth capability is constrained by available air pressure and hydraulic losses within the tubing system, while reliance on a surface compressor may limit deployment in remote environments. Addressing these aspects through technological improvements will further enhance the system’s applicability and robustness.

Overall, the PDA-MLS shows strong potential as a promising multilevel groundwater monitoring technology for multilevel groundwater monitoring, particularly in complex hydrogeological settings where conventional methods are inadequate. With continued development and integration into advanced monitoring frameworks, the system may significantly contribute to improving groundwater characterization and contaminant assessment practices.

Future research should focus on consolidating and extending the capabilities of the PDA-MLS through both experimental validation and technological development.

First, benchmarking studies should be conducted against conventional low-flow pumping systems operating under packer isolation in wells without floating contaminants. Such comparisons would enable a quantitative evaluation of relative performance with respect to flow stability, stabilization time, and sample representativeness.

Second, efforts should be directed toward extending the operational depth of the system beyond 90 m. This may be achieved by combining higher-pressure actuation with optimized tubing configurations and low-loss valve systems, supported by detailed experimental assessment of pressure-loss budgets.

Third, the development of compact and portable actuation systems—such as cartridge-based gas units or battery-powered micro-compressors—would enable deployment in remote or off-grid environments. These solutions should also consider intrinsic safety requirements for operation in hazardous or contaminated sites.

Fourth, the integration of embedded micro-sensors (e.g., pressure, temperature, and electrical conductivity) within each sampling unit represents a key advancement. Such integration would allow real-time monitoring of purge conditions, automated stabilization detection, and continuous data logging, thereby improving both sampling efficiency and data quality.

Fifth, long-term durability testing should be undertaken to evaluate material performance under repeated operational cycles, including membrane fatigue, elastomer aging, and resistance to chemical exposure. Accelerated laboratory testing combined with repeated field deployments would provide valuable insights into system longevity and maintenance requirements.

Finally, the implementation of multi-well monitoring arrays and synchronized sampling strategies should be explored to generate three-dimensional, depth-resolved datasets. Such datasets would significantly enhance the characterization of fractured and karst aquifers and support the development of advanced hydrogeological models and decision-support tools.

## Figures and Tables

**Table 1:** Summary of depth-discrete groundwater sampling tools, including construction materials, extraction methods, applicable aquifer types, and maximum effective sampling depths. References correspond to the original studies documenting tool design, performance, or field applications. (*) Category of samplers including the device proposed in this study.

Multilevel Sampler	Sampler Materials	Method	Aquifer Type	Sampling Depth	Ref.
Sealed multiport sampling (SMPS)	PTFE tubes with water-inlet screens	Suction filtration and extraction (peristaltic pump)	Unconsolidated aquifers	<10 m	[23,24,25]
Continuous Multichannel Tubing (CMT)	7-channel HDPE tubing	Motor-driven inertial pump	All	<79 m	[13]
		Manual operation	All	<30 m	[9,10]
		Embedded inertial pump	All	<40 m	[13]
Groundwater profile sampler (GPS)	Fiberglass probes inside a screened well point	Suction filtration and extraction	Coastal aquifers	<9.0 m	[26]
Permanent multilevel sampling system	Nylon tubes with water-inlet ports connected to a cone-tip	Suction filtration and extraction	Coastal sandy aquifers	<4.5 m	[27]
Pressurized Driving-Down Air Multilevel Sampler (PDA-MLS) (*)	Stainless steel, PTFE, nylon/polyurethane tubing	Pressurized-air (≤1.0 MPa) pump-samplers	All	<50 m	[28]
Flexible Liner Underground Technologies (Water FLUTe^®^)	Urethane-coated nylon liner with mesh sampling ports for rock borings	Gas-driven pump-tube system (long U-shaped tube)	Consolidated or fractured aquifers	<150 m	[14]
Hydraulic Profiling Tool—Groundwater Sampler (HPT-GWS)	Iron probe with sampling ports and EC transducer	Direct-push method using percussion or hydraulic hammer	Alluvial (unconsolidated) aquifers	<30 m	[29]

**Table 2:** Field operating conditions applied during PDA-MLS deployment at well PZ4.

Parameter	Symbol/Variable	Value (Unit)
Well ID	—	PZ4
Ground elevation	—	67 m ASL
Water table depth	—	41.7 m BGL
Floating NAPL thickness	—	0.20–0.30 m
Depth of upper sampler	S_1_	57.5 m BGL
Depth of lower sampler	S_2_	60.0 m BGL
Vertical spacing between samplers	—	2.5 m
Compressor working pressure	P	0.8 MPa (max 1.0 MPa)
Pressure tubing material	—	Polyurethane (PU SH98)
Water tubing material	—	Polyamide (Rilsan PA11)
Tubing internal diameter	d	2 mm
Gas injection interval	Δt	15 s
Sampler chamber volume	Sc	42.6 mL
Estimated inflow discharge	$q_{in}$	≈166 mL min^−1^
Estimated outflow discharge	$q_{out}$	≈162 mL min^−1^
Observed field discharge	$q_{obs}$	≈145 mL min^−1^
Purge duration	—	10 min
Collected volume per sampler	—	4 × 1000 mL
Laboratory analyses	—	EPA 8260D + hydrochemical panel
Transport and storage	—	Glass vials, portable freezer

**Table 3:** Selected chemical indicators supporting sampling-integrity assessment in groundwater samples collected with the PDA-MLS.

Parameter (Unit)	S_1_ (57.5 m)	S_2_ (60.0 m)
Temperature (°C)	17	17
Electrical conductivity (µS/cm)	724	738
pH (—)	7.9	7.4
Trichloroethylene, TCE (µg/L)	0.10	0.06
Tetrachloroethylene, PCE (µg/L)	<0.10	0.10
Vinyl chloride (µg/L)	<0.01	<0.01
1,2-Dichloroethylene (µg/L)	<0.10	<0.10
Benzene (µg/L)	0.20	1.10
Ethylbenzene (µg/L)	<0.10	<0.10
Toluene (µg/L)	0.10	0.10
m,p-Xylene (µg/L)	<0.10	0.20
Total halogenated organics (µg/L)	0.21	2.00

Notes: Values reflect ARPA Puglia laboratory determinations (EPA 8260D for VOCs) from the field campaign reported in the manuscript. Sampling depths refer to the top of each sampler module.

**Table 4:** Comparative performance of the PDA-MLS and conventional groundwater sampling methods. Qualitative comparison based on published literature, operational considerations, and field observations.

Criterion	PDA-MLS (Low-Flow Pressurized Sampler)	Low-Flow Pump	Bailer	Packer-Isolated System
Hydraulic Disturbance	Minimal disturbance; pressure-driven extraction preserves vertical stratification and avoids mixing	Moderate disturbance due to continuous pumping and drawdown [41,42,43]	High disturbance during insertion and retrieval; induces mixing [44,45]	Low disturbance locally, but may alter hydraulic conditions during isolation [42,46]
NAPL Interaction	Capable of sampling below floating NAPL without disturbing the layer	Risk of downward displacement or entrainment of NAPL [42,47]	Cannot operate effectively in presence of floating NAPL [42,45]	Limited ability to isolate zones below NAPL [42]
VOC Preservation	High preservation; inert materials and no aeration minimize volatilization	Moderate; degassing and volatilization during pumping may occur [41,44]	Low; aeration and volatilization significant [44,45]	Moderate; dependent on sealing efficiency and system integrity [42]
Chemical Inertness	High; PTFE and stainless steel minimize sorption and leaching	Moderate; tubing materials may affect sample chemistry [42,43]	Low to moderate; possible contamination from sampler materials [45]	Moderate; depends on packer composition and materials [42]
Operational Depth Capability	High; stable performance up to ~60–90 m under pressurized conditions	Moderate; efficiency decreases with increasing depth [41,42]	Limited control at depth; inefficient in deep wells [45]	Variable; depends on system design and installation [42,46]
Mechanical Reliability	High; suitable design tolerates clogging, irregular boreholes, and NAPL presence	Moderate; sensitive to clogging and maintenance [42,43]	High simplicity but low operational control [45]	Complex; sensitive to installation and sealing conditions [42,46]
Ease of Installation and Use	Simple, modular, automated operation; no specialized training required	Moderate; requires calibration and skilled operation [42,43]	Very simple but lacks control and reproducibility [45]	Complex; requires skilled personnel and careful setup [42]
Sampling Resolution (Depth Discrete)	High; simultaneous multilevel sampling	Limited; typically single-depth sampling [42,43]	Limited; discrete but not simultaneous [45]	High; depth-discrete but operationally complex [42,46]
Suitability for Fractured Aquifers	Good; preserves hydraulic gradients and avoids disturbance of fracture flow	Limited; pumping may alter flow conditions [41,44]	Poor; induces mixing and loss of stratification [45,46]	Moderate; depends on effective sealing [46]
Automation and Repeatability	High; programmable cycles ensure reproducibility	Moderate; operator-dependent variability [42,43]	Low; manual operation introduces variability [45]	Low to moderate; manual control required [42]

## Data Availability

The data presented in this study are available from the corresponding author upon reasonable request.

## Abbreviations

The following abbreviations are used in this manuscript:

ARPA	Regional Environmental Protection Agency
ASL	Above Sea Level
BGL	Below Ground Level
CMT	Continuous Multichannel Tubing
EC	Electric Conductivity
DNAPL	Dense Non-Aqueous Phase Liquid
FLUTe	Flexible Liner Underground Technologies
GPS	Groundwater Profile Sampler
HPT—GWS	Hydraulic Profiling Tool—Groundwater Sampler
PCE	Tetrachloroethylene
PDA-MLS	Pressurized Driving-Down Air Multilevel Sampler
PTFE	Polytetrafluoroethylene (Teflon)
SMPS	Sealed Multiport Sampling
VOC	Volatile Organic Compound
